# Restricted Ketogenic Diet Therapy for Primary Lung Cancer With Metastasis to the Brain: A Case Report

**DOI:** 10.7759/cureus.27603

**Published:** 2022-08-02

**Authors:** Athanasios E Evangeliou, Martha G Spilioti, Despoina Vassilakou, Fotini Goutsaridou, Thomas N Seyfried

**Affiliations:** 1 Fourth Department of Pediatrics, Division of Child Neurology and Metabolism, Papageorgiou Hospital, Aristotle University of Thessaloniki, Thessaloniki, GRC; 2 First Department of Neurology, AHEPA Hospital, Aristotle University of Thessaloniki, Thessaloniki, GRC; 3 Third Department of Internal Medicine, Papageorgiou Hospital, Aristotle University of Thessaloniki, Thessaloniki, GRC; 4 Department of Radiology, Papageorgiou Hospital, Thessaloniki, GRC; 5 Biology, Boston College, Boston, USA

**Keywords:** lipidemic profile, dietary energy restriction, brain metastasis, primary lung cancer, ketogenic diet

## Abstract

A high-fat and low-carbohydrate diet was administered as a complementary and alternative therapy to a 54-year-old man suffering from non-small-cell lung cancer (NSCLC) with brain metastasis. Three months after the cessation of chemotherapy and radiotherapy, a ketogenic diet (KD) was initiated. This approach was an attempt to stabilize the disease progression after chemotherapy and radiotherapy. Computed tomography following radiation and chemotherapy showed a reduction in the right frontal lobe lesion from 5.5 cm × 6.2 cm to 4 cm × 2.7 cm, while the mass in the upper-right lung lobe reduced from 6.0 cm × 3.0 cm to 2.0 × 1.8 cm. Two years after KD initiation and without any other therapeutic intervention, the right frontal lobe lesion calcified and decreased in size to 1.9 cm × 1.0 cm, while the size of the lung mass further decreased to 1.7 cm × 1.0 cm. The size of the brain and lung lesion remained stable after nine years of KD therapy. However, dyslipidemia developed after this time which led to the discontinuation of the diet. No tumor relapse or health issues occurred for two years after the discontinuation of the diet. This case report indicates that the inclusion of ketogenic metabolic therapy following radiation and chemotherapy is associated with better clinical and survival outcomes for our patient with metastatic NSCLC.

## Introduction

Despite progress in chemotherapy, radiation, and surgical procedures, malignant cancer continues to be a leading cause of death globally. Therefore, there is a need to develop new therapeutic procedures that are more effective than current approaches. Some factors in the pathophysiology of the malignant cells can possibly suggest improved therapeutic options.

Malignant cancer cells express particular metabolic characteristics that distinguish them from healthy cells. Specifically, most cancer cells lack metabolic versatility due to mitochondrial abnormalities and are largely dependent on glucose for energy according to the Warburg theory of cancer [1,2]. This common phenotype of tumor cells contrasts with that of normal brain cells, which derive energy from ketone bodies when glucose becomes limited [1]. Consequently, it makes sense to question the fate of cancer cells when their primary glycolytic fuel is reduced.

A simple approach is to inhibit glycolysis while increasing circulating ketone bodies. The high-fat/low-carbohydrate ketogenic diet (KD) is a good option for patients with brain cancer. The KD might also limit the availability of glutamine [3]. Glucose and glutamine together provide the majority of energy needed for the growth of tumor cells [4].

Ketogenic metabolic therapy (KMT) is becoming recognized as an effective complementary or alternative therapeutic strategy for managing cancer [5-7]. Nebeling et al. found that a KD consisting of medium-chain triglycerides provided long-term management of pediatric astrocytomas while enhancing the nutritional status of patients [8]. The findings in human pediatric astrocytoma were confirmed in an experimental mouse model of astrocytoma using a lard-based rodent KD [9].

Seyfried et al. also demonstrated that dietary energy restriction and restricted KDs that lower blood glucose while elevating blood ketone bodies have anti-tumor and anti-angiogenic effects in several experimental mouse models of brain tumors [10]. Further support for this metabolic cancer therapy has been obtained for human glioblastoma [11-13]. In this study, we describe the favorable course of a patient diagnosed with non-small-cell lung cancer (NSCLC) with neuroendocrine features and brain metastasis who was administered a KD for three months following the completion of radiation and chemotherapy.

## Case presentation

**Figure 1:**
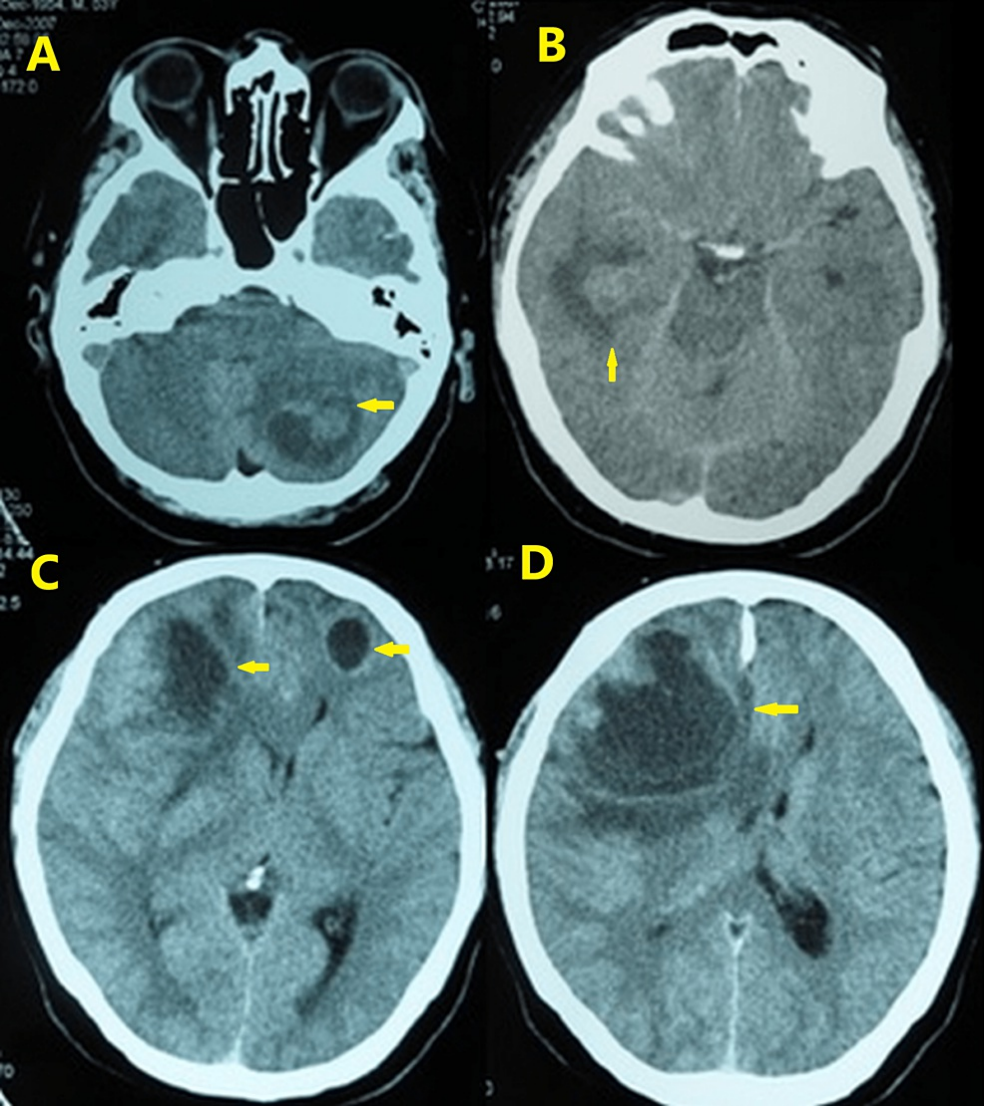
Brain computed tomography on December 31, 2007. Post-contrast medium axial images displaying intracranial metastases as low-density mass-like lesions with heterogeneous or ring-like enhancement and perifocal edema in the left cerebellar hemisphere (A), right temporal lobe (B), and bilateral frontal lobes (C, D). The size of the metastatic lesion in the right frontal lobe (D) is 5.5 cm × 6.2 cm with perifocal vasogenic edema, mass-effect toward the frontal horn of the right lateral ventricle, and midline shift to the left.

**Figure 2:**
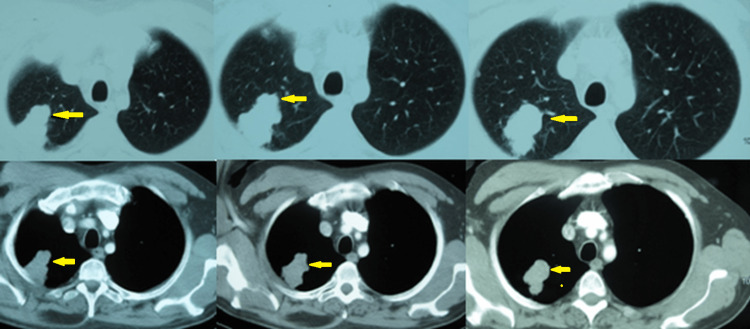
Chest computed tomography on December 31, 2007. Chest axial images (soft tissue and lung windows) revealing a mass of 6 cm × 3 cm in the right upper lobe (apical and posterior segment) of the lung without lymph nodes and some micronodules in the upper and middle lobe.

**Figure 3:**
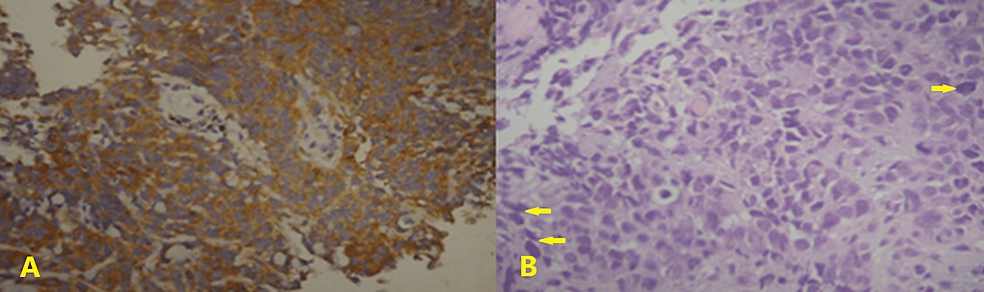
Pathology of the primary tumor. A: Immunostaining for chromogranin AX200. Positive cytoplasmic staining indicating the neuroendocrine differentiation of neoplasmatic cells (NSCLC with endocrine features). B: Hematoxylin-eosin (original magnification ×200). Part of the primary tumor revealing neuroendocrine-type cells with high nuclear-to-cytoplasm ratio, pleomorphism, and solid growth pattern (arrows), features of high-grade malignant neuroendocrine neoplasm (NSCLC). NSCLC: non-small-cell lung cancer

A 54-year-old right-handed man presented with headache, dizziness, gait ataxia, vomiting, and blurred vision one week before admission on December 30, 2007. A computed tomography (CT) scan of the brain obtained in the emergency room revealed multiple metastatic lesions in the left cerebellum, right temporal lobe, and frontal lobes bilaterally (Figure 1). The patient’s medical history was unremarkable, but he had smoked heavily (four to five packs/day) for 36 years. Imaging analysis was performed to reveal the primary lesion. A chest CT scan revealed a mass of 6.0 cm × 3.0 cm in the upper-right lobe (Figure 2), and the lung biopsy was compatible with NSCLC with neuroendocrine features (Figure 3). The staging images did not show any other lymph node or metastatic disease.

The patient received whole brain radiation therapy (3,000 Gy over 10 daily sessions) from January 24, 2008, until February 7, 2008. No radiation therapy was performed on the lungs. Paclitaxel/carboplatin chemotherapy was provided from March 3, 2008, until June 17, 2008. The patient received six cycles of chemotherapy with paclitaxel (175 mg/m^2^) and six cycles of carboplatin AUC every 21 days and showed good tolerance. A KD was initiated in June 2008 during the follow-up phase after chemotherapy and radiation were completed. At this point, chemotherapy and radiation had no effect on reducing primary and secondary tumor lesions.

The KD was administered as a complementary alternative therapy in an attempt to stabilize disease progression. The diet was started with a 1:1 ketogenic ratio and gradually reached 3:1. The diet was adapted to Mediterranean diet patterns due to the patient’s aggravated condition from the disease and chemotherapy to avoid additional side effects. This diet pattern is milder than the classic North American-Northwestern European KD. An example of the diet applied in its final form (3:1) is shown in Table 1.

**Table 1:** Daily restricted ketogenic diet plan. The patient chose three meals each day from five meal choices (meals A-E).

	Serving size (g)	Fat (g)	Protein (g)	Carbohydrate (g)	Calories (kcal)
Meal A					
Beef or pork	87	14.5	20.3		212
Broccoli	26		0.3	0.8	7
Olive oil	62	49.6			446
Total		64.1	20.6	0.8	665
Meal B					
Chicken	87	14.5	20.3		212
Cabbage	26		0.3	0.8	7
Olive oil	62	49.6			446
Total		64.1	20.6	0.8	665
Meal C					
Tuna in water	87	14.5	20.3		212
Cabbage	26		0.3	0.8	7
Olive oil	26	49.6			446
Total		64.1	20.6	0.8	665
Meal D					
Two eggs		10	14		146
Bacon NIKac	so	14.5	7	1	166
Olive oil	49	39.2			353
Total		63.7	21	1	664
Meal E					
Sardines	101	14.4	20.2		210
Cabbage	26		0.3	0.8	7
Olive oil	62	49.6			446
Total		64	20.5	0.8	664
Average daily total*		192	62	2.5	1,995

**Figure 4:**
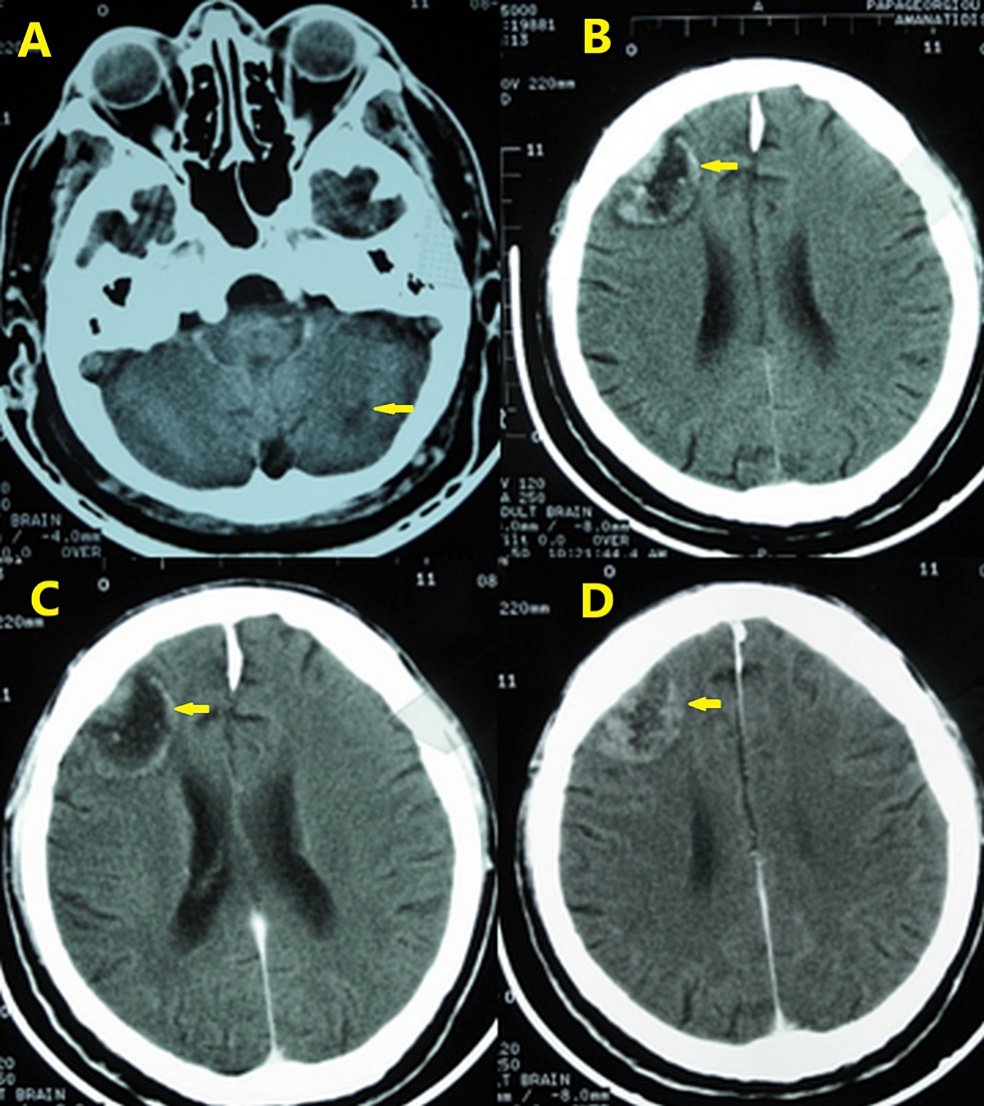
Brain computed tomography scan on July 8, 2008. Axial CT images plus contrast medium. In the left cerebellar hemisphere, there is a small low-density lesion without enhancement post-contrast medium and no perifocal edema or mass effect (A). Reduction in the size of the right frontal lobe lesion from 5.5 cm × 6.2 cm to 4 cm × 2.7 cm with no perifocal edema or mass effect on the right ventricle (B-D). No left lesions in the left frontal lobe and the right temporal lobe.

**Figure 5:**
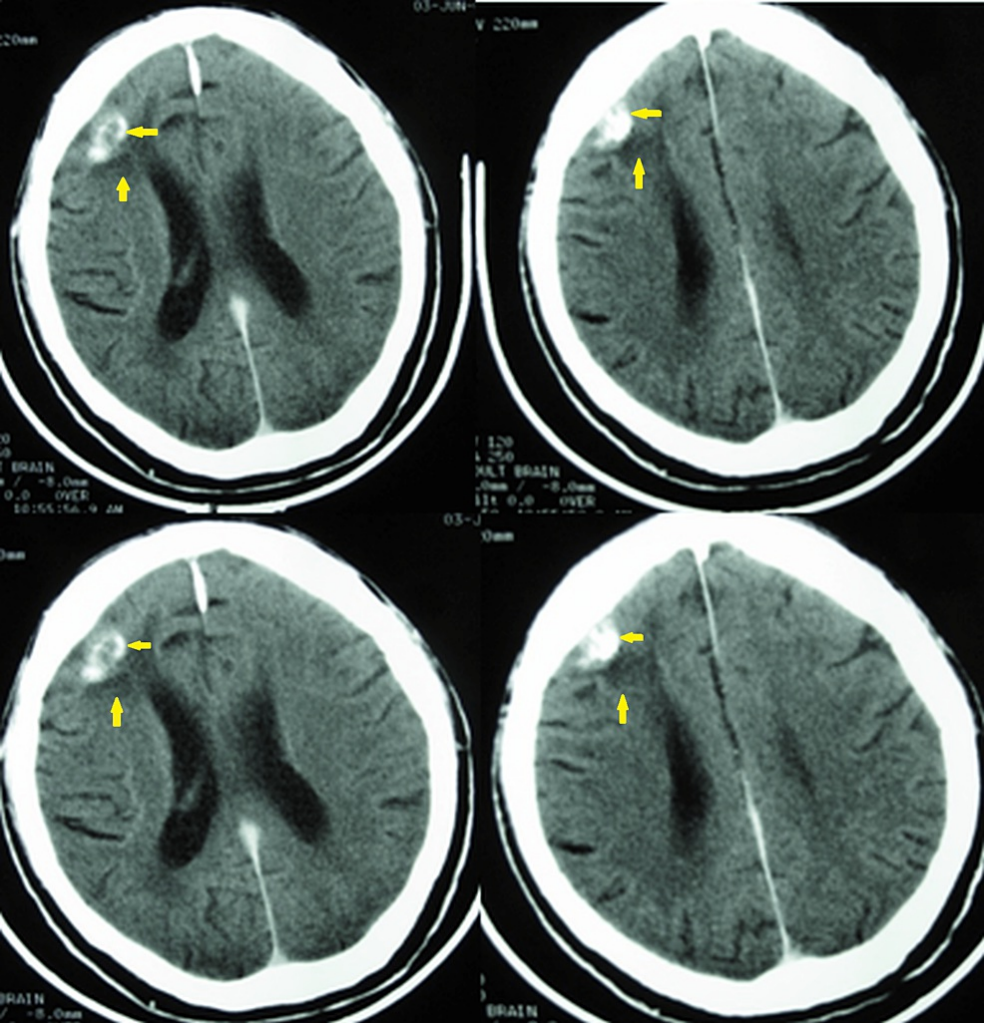
Brain computed tomography scan on October 7, 2009. Axial post-contrast images revealing a further reduction in the size of the right frontal lobe lesion to 1.9 cm × 1 cm. Images display focal calcification at the site of previous metastasis with no enhancement or perifocal edema (horizontal arrows). Low-density area behind the calcification with little expansion of the ipsilateral lateral ventricle body due to gliosis (vertical arrows).

**Figure 6:**
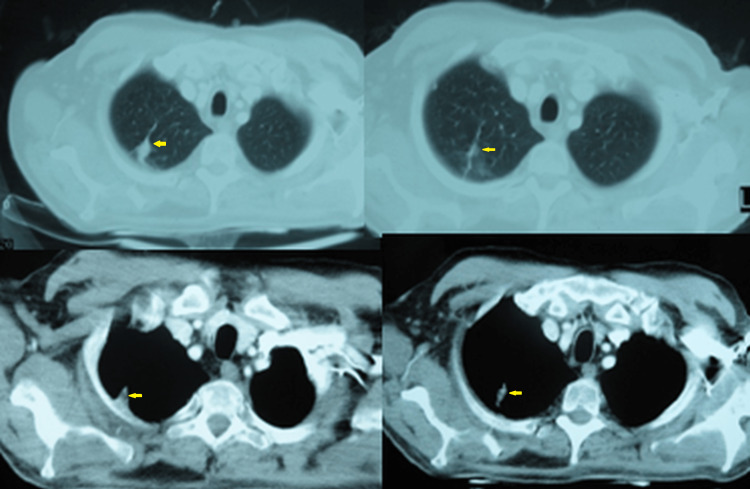
Chest computed tomography scan on October 7, 2009. Axial images (lung and soft-tissue density windows) showing a reduction of the right upper lobe mass.

**Figure 7:**
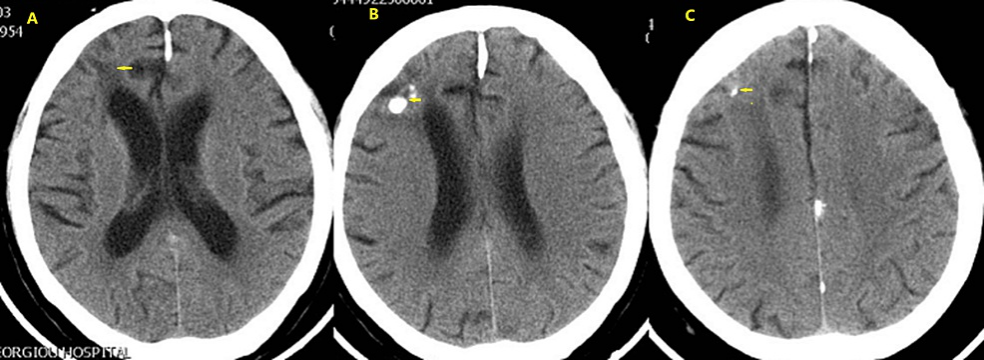
Brain computed tomography scan on January 7, 2011. Post-contrast axial images revealing a low-density area in front of the right frontal horn (A) and calcification in the right frontal lobe with no change in lesion dimensions and no enhancement (B, C). No other focal lesions are revealed.

**Figure 8:**
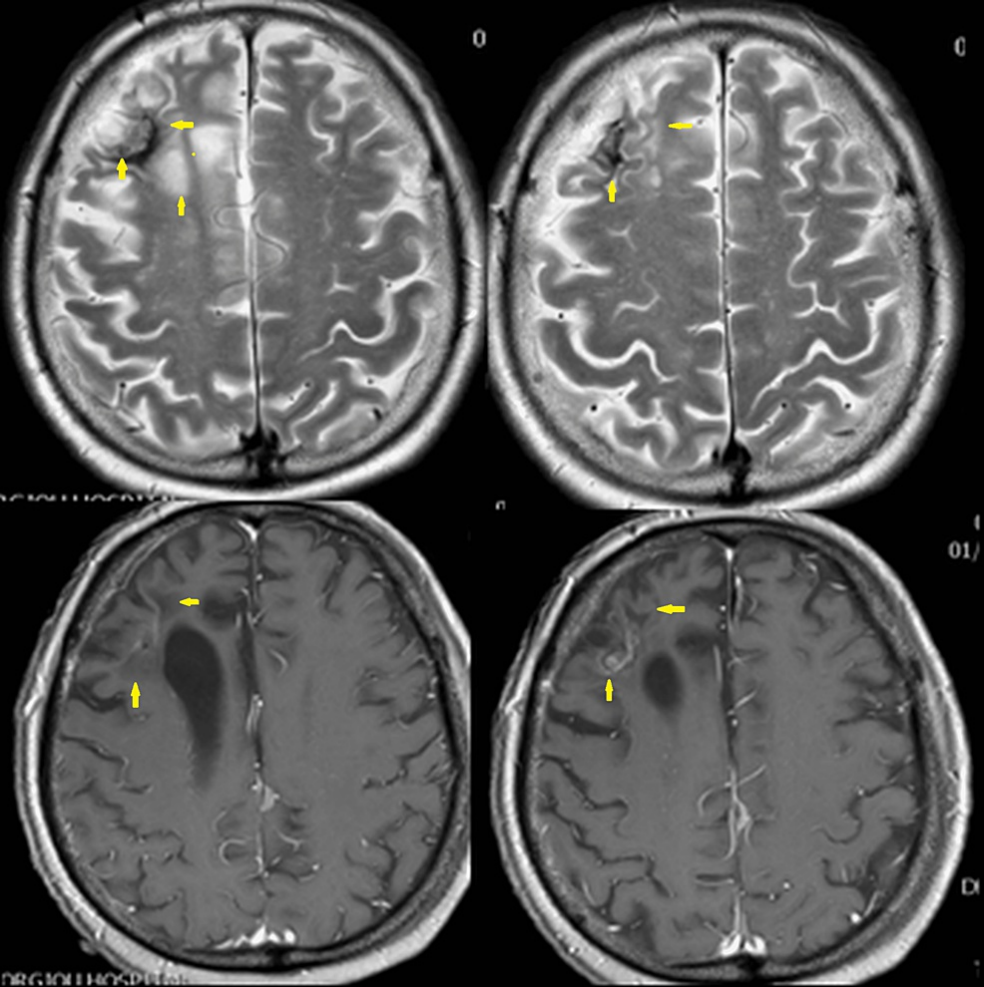
Brain magnetic resonance imaging on January 10, 2018. Gliotic and calcified right frontal lobe lesions. No other lesions are revealed.

**Figure 9:**
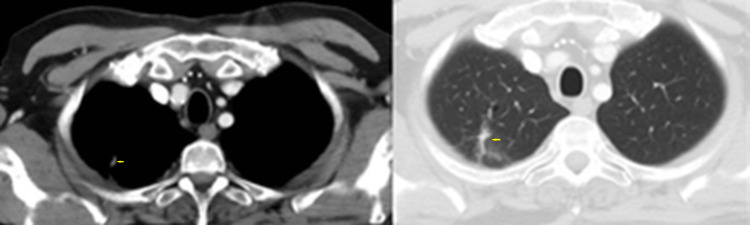
Chest computed tomography scan on June 13, 2018. Stable findings of the lungs.

Figures 4-9 show the first brain CT scan after completion of radiation and chemotherapy, as well as the development until the last follow-up brain CT scan, chest CT scan, and brain magnetic resonance imaging (MRI) scan 10.5 years after the KD initiation. No serious adverse effects were noticed during the course of the KD therapy. The patient developed kidney stones in June 2011, which were easily removed through lithotripsy.

During the course of the KD therapy, glucose levels remained at 60 to 70 mg/dL (3.3-3.9 mmol/L), while the ketone levels were elevated to about 2 mmol/L. These glucose and ketone values produced a glucose-ketone index (GKI) of approximately 1.8, indicating a high therapeutic level of ketosis. The patient’s body weight remained relatively stable during the 10-year KD treatment period (his initial weight was 87 kg, and his final weight was 82 kg). Moreover, during the same period, the patient’s lipidemic profile was stable without the appearance of any dyslipidemia. Table 2 provides a summary of the tumor progression, type of KD, and daily caloric intake in relation to ketone, glucose, lipid, and weight. The KD was terminated 10 years after diet initiation (2018) as the patient developed dyslipidemia (cholesterol >280 mg/dL, triglycerides >300 mg/dL). It has now been almost four years since the patient stopped the KD, and he remains healthy with no evidence of tumor recurrence.

**Table 2:** Influence of the ketogenic diet on metastatic tumor growth and circulating energy metabolites in a patient with non-small-cell lung cancer. C-CT: chest computed tomography; B-CT: brain computed tomography; B-MRI: brain magnetic resonance imaging; Glu: fasting glucose levels (mg/dL); KB: ketones in blood (mmol/L); Cho: cholesterol (mg/dL), HDL-cho: high-density lipoprotein cholesterol (mg/dL); LDL-cho: low-density lipoprotein cholesterol (mg/dL); Tri: triglycerides (mg/dL); W: patient’s weight; DCI: daily calorie intake; KDR: ketogenic diet ratio represented as fats:carbohydrates + protein

Date	C-CT (cm)	B-CT (cm)	B-MRI	Glu	KB	Cho	HDL-cho	LDL-cho	Tri	W	DCI	KDR
08.07.08	2 × 1.7			86–90	0.6	220	40	120	170	76	2,275	1:1
12.09.08	2 × 1.6			61–71	0.9	180	50	100	150		2,293	2:1
26.09.08		3.1 × 2		86–90	0.8						2,293	3:1
05.03.09	1.7 × 1.5	2 × 1.5		71–73	1.5						2,000	3:1
03.06.09	1.7 × 1.5	2 × 1.5		65	2.1						1,700	3:1
07.10.09	1.7 × 1	1.9 × 1		71	2.1						1,700	3:1
31.05.10	1.7 × 0.8	1.8 × 1		75	2.0	180	55	100	160		2,200	3:1
15.10.10		1.8 × 1		65	2.0						2,200	3:1
07.01.11	1.7 × 0.8	1.8 × 1		70	1.9						2,100	3:1
06.10.11		1.8 × 1		60	2.1						2,000	3:1
15.02.12	1.7 × 0.8	1.8 × 1		70	2.1						2,100	3:1
13.06.18	1.7 × 0.8			70	2.1							
08.07.18			1.7 × 0.8	70	2.0	280	45	140	300		2,100	3:1

## Discussion

Previous case reports have shown that the KD can be effective in managing primary brain cancer in children and adults [8-12]. However, to our knowledge, no prior case studies have described a therapeutic effect of a KD against metastatic or secondary brain cancer. We attempted to address the cause of the favorable disease course in our patient. The favorable response to therapy in this patient could have been an effect of conventional therapy, the KD therapy, or a combination of these effects.

NSCLC is a highly aggressive pulmonary neuroendocrine tumor, and affected patients generally have a significantly worse prognosis than patients with large-cell carcinomas, even in stage 1 disease [14,15]. Moreover, little has been established regarding a standard treatment strategy for this type of advanced clinical cancer. Growth management is not generally observed after chemo and radiation therapy [16,17]. Remarkably, the patient’s tumor growth, including brain metastasis, was stabilized and even diminished about one year after completion of the standard treatment and long after. The only therapeutic intervention during this period was the KD. Therefore, it is possible that the diet was partly responsible for the patient’s improvement.

The above beneficial effect of the KD is also supported by the fact that no effect on the disease course was observed throughout the latest period when the patient was receiving conventional therapy. It is important to mention that the low-normal glucose levels could have also contributed in part to the restricted tumor growth in our patient. Previous studies in mice and humans with brain cancer have shown that low blood glucose levels are associated with reduced brain tumor growth rate and better survival, while elevated glucose or hyperglycemia contributes to rapid tumor growth and poor patient survival [9,18]. Elevated blood glucose is also a risk factor for breast cancer and NSCLC [19]. It is likely that the combination of both reduced glucose levels and elevated ketone levels contributed to tumor management, as described previously [8,19].

Another interesting point is the lipidemic profile of the patient. The patient developed dyslipidemia only after 10 years on the 3:1 KD without evidence of tumor recurrence. At first glance, this observation could be correlated to the fact that the tumor was no longer active. However, further studies will be needed to determine the origin of dyslipidemia following long-term treatment of cancer patients with KMT.

## Conclusions

This case report gives hope that the KD might improve clinical outcomes for some patients with NSCLC. The tumor shrinkage, survival time, spectacular improvement in physical condition, and the restoration of daily attitudes are encouraging for further application of this therapeutic approach to larger numbers of patients.
